# Labor Status at Delivery and Lung Function in Extremely Prematurely Born Young Adults

**DOI:** 10.1002/ppul.27440

**Published:** 2024-12-16

**Authors:** Sean Armstrong, Christopher Harris, Mohadeseh Kazemi, Alan Lunt, Janet Peacock, Anne Greenough

**Affiliations:** ^1^ Neonatal Intensive Care Centre King's College Hospital NHS Foundation Trust London UK; ^2^ Department of Epidemiology Geisel School of Medicine at Dartmouth, Dartmouth College Hanover New Hampshire USA; ^3^ Women and Children's Health, School of Life Course Sciences, Faculty of Life Sciences and Medicine King's College London London UK

**Keywords:** infant, lung function, newborn, prematurity, young adults

## Abstract

**Background:**

There has been conflicting evidence regarding the impact of mode of delivery on respiratory outcomes in later childhood and adulthood. It is possible labor status, rather than mode of delivery, influences later respiratory morbidity. We hypothesized that extremely premature infants born to mothers in labor would have better lung function at follow‐up than those born to mothers not in labor.

**Methods:**

We reviewed data from the United Kingdom High‐Frequency Oscillation Study. Lung function testing was performed on young people aged 16–18 years born before 29 weeks of gestation. Linear mixed models were used to adjust lung function for maternal and neonatal factors and for the clustering due to multiple births.

**Results:**

One hundred and fifty subjects underwent lung function testing. Young adults born to mothers in labor had better mean Forced Expiratory Flow_75_ (FEF_75_) compared to those born to mothers not in labor (adjusted difference 0.50 [95% CI: 0.02, 0.99]). Similar significant differences were noted in FEF_50_ (0.45 [−0.05, 0.85]), and FEF_25‐75_ (0.53 [0.05, 1.01]).

**Conclusion:**

Our study demonstrates that amongst individuals born very prematurely, those whose mothers were in labor before delivery had better small airway function at 16–19 years of age.

## 1 Introduction

During labor, infants undergo a series of physiological adaptations for postnatal life—a process which may be impaired in infants delivered by elective cesarean section (CS) [1, 2]. There are significant differences in hormone levels between infants born following labor compared to those born to mothers not in labor—reduced levels of neuroendocrine hormones have been shown in infants born by CS [3, 4]. Newborn levels of maternal hormones (e.g., progesterone) fall faster in infants born following maternal labor compared to in those delivered by CS [5]. Those changes, following CS delivery, may alter the HPA axis in newborns—as evidenced by increased pain responses in the newborn period [6]. Vaginal deliveries are associated with higher levels of immunological activation compared to CS deliveries [7]. This may be facilitated by exposure to maternal microorganisms—with immune activation by the microbiome response—during labor and vaginal delivery [8, 9]. Studies have demonstrated that infants born by cesarean section have increased rates of respiratory complications in the immediate neonatal period [10]. It has been suggested that infants born after maternal labor establish an FRC earlier and thus may have better neonatal lung function than infants born to mothers not in labor [11]. Early studies suggested a link between cesarean section at term and wheeze or asthma symptoms in later childhood [12, 13, 14]. Other studies have failed to show any significant correlation [15, 16, 17, 18, 19, 20, 21]. None of these studies, however, examined the labor status of these women, and only two studies have analyzed emergency and elective cesarean sections separately [20, 21]. One study showed no difference in zFEV_1_ scores in school‐going children born by elective cesarean section or vaginal delivery (*p* = 0.8) [20]. Another study found a significant difference in the percentage of 3 year olds born by emergency versus elective cesarean section who had an active wheeze (4.79% vs. 3.45%, *p* = 0.044) [21]. Furthermore, all of the studies focused on term infants; three studies included a small number of late preterm infants, without analyzing them separately [15, 16, 21]. In a prospective cohort study of 6128 children, the effect of elective and emergency cesarean section with wheezing outcomes were similar, but only elective cesarean section was associated with a higher fractional exhaled nitric oxide (FeNO) level, suggesting increased airway inflammation [22]. It is possible that the inclusion of mothers who were in labor in any cesarean section group may confound efforts to establish causal links to childhood respiratory morbidities.

Children and young adults born preterm have poorer lung function compared to term controls [23, 24]. This effect persists into adulthood [25]. There is a paucity of research determining whether maternal labor status affects long‐term respiratory outcomes in prematurely born infants. The aim of this study then was to determine whether mode of delivery or if the mother was in labor before delivery affected lung function in young adults born extremely prematurely. We hypothesized that lung function in young adults born prematurely to mothers who were in labor would be significantly better than in young adults born prematurely to mothers who were not in labor.

## 2 Materials and Methods

Young people who had been recruited into the United Kingdom Oscillation Study (UKOS) were invited to King's College Hospital NHS Foundation Trust to undergo lung function and exercise testing and to complete a health questionnaire when they were aged between 16 and 19 years of age [26]. Participants gave written consent to participate in the study, and their parents had previously given written consent to participation in the UKOS trial. Ethical approval for UKOS was granted by the Thames Multicentre Research Ethics Committee [27] and for the follow‐up study by the North‐East–Tyne & Wear South Research Ethics Committee (16/NE/0314).

Participants underwent respiratory testing in accordance with American Thoracic Society and European Respiratory Society standards [28, 29, 30, 31, 32]. Spirometry was used to measure Forced Expiratory Flow at 75% (FEF_75_), Forced Expiratory Flow at 50% (FEF_50_), Forced Expiratory Flow at 25% (FEF_25_), Forced Expiratory Flow at 25%–75% (FEF_25‐75_), Forced Vital Capacity (FVC), and Peak Expiratory Flow (PEF). Plethysmography measurements of Functional Residual Capacity (FRC_PLETH_), Total Lung Capacity (TLC_PLETH_), and Residual Volume (RV_PLETH_) were made. In addition, Functional Residual Capacity (FRC_He_) by helium dilution, impulse oscillometry at 5 Hz and 20 Hz, Diffusion Capacity of Carbon Monoxide (DLCO, DLCO/VA) and Lung Clearance Index (LCI) were assessed.

Spirometry, plethysmography, impulse oscillometry, DLCO, and FRC by helium dilution were measured using Vyair lung function equipment. Lung clearance index was assessed using the SF_6_ (Innocor) multi‐breath washout technique. This closed‐circuit technique uses 0.2% Sulfur Hexafluoride and is felt to be more advantageous over traditional N_2_ testing as there is no back‐wash into the lungs [33]. The wash‐in/wash‐out maneuvers can be performed at any time in the breathing cycle. Recently, reference ranges for SF_6_ LCI testing have been made available [34]. Lung function results were converted to z‐scores using Global Lung Initiative (GLI) reference equations or appropriate reference equations for those lung measurements not included in the GLI reference equations [35, 36, 37, 38]. A shuttle sprint test was used to assess exercise capacity [39]. Multilevel Shuttle Runs have been established as a standard of Cardiopulmonary Exercise Testing (CPET) in child‐ and adulthood [40, 41].

Participants completed a questionnaire regarding their respiratory symptoms, whether they had a current diagnosis of asthma and how much exercise they undertook on a weekly basis. Smoking status of the participants was recorded as “yes” if either they were a self‐reported smoker or had a salivary cotinine level greater than 15 ng/mL. Puberty was previously assessed when the participants were 11–14 years of age. More than 90% of children were found to be of Tanner stage 2.

FEF_75_ was the primary outcome of this study to remain consistent with the primary outcome of other studies of this cohort [42]. Small airway changes seen in respiratory disease are reflected by increases in airway (< 2 mm) resistance and a reduction in flow seen in the terminal portion of the spirogram [43, 44]. This can be demonstrated either through FEF_75_ or FEF_25‐75_ testing [45]. Demographic factors, lung function, exercise and respiratory symptoms were assessed for statistical significance first by delivery mode, and then by maternal labor status, using mixed models to adjust for the nonindependence of multiple births [46] (continuous data) or logistic regression with a robust standard error [47] (binary outcomes). The effects of delivery mode and maternal labor on lung function were analyzed using a similar mixed model approach but including the following confounders: maternal smoking in pregnancy, gestational age at birth, birthweight z‐score, administration of surfactant (yes/no), and participant age at lung function assessment. Analysis of binary outcomes (symptoms/medication) used logistic regression with a robust standard error and adjusted for birthweight z‐score. Analysis was performed using Stata v 18 and R.

## 3 Results

One hundred and fifty UKOS participants returned for lung function testing at 16–19 years. The participants who were assessed at age 16–19 years had a higher mean birthweight and gestational age and their mothers were more likely to be white and not to have smoked in pregnancy compared to the whole UKOS cohort. They were also more likely to have had a major cranial ultrasound abnormality and a pulmonary hemorrhage (Table S1).

There were significant differences in mean birthweight, birthweight z‐score, gestational age, and maternal smoking status between those born by vaginal delivery and those born by Cesarean Section (Table 1). There were statistically significant differences in birthweight, birthweight z‐score, gestational age, and maternal smoking in pregnancy between those born after maternal labor compared to those whose mothers were not in labor (Table 2). Most lung function results were not statistically different between infants been born by vaginal delivery or cesarean section, except FEF_75_ (Table 3).

**Table 1:** Demographics by type of delivery.

	Vaginal delivery	Cesarean	P value
**Number**	**66**	**84**	
Female	36 (55%)	44 (52%)	0.790
Birthweight (gram)	919 (225)	889 (214)	0.280
Birthweight z‐score	−0.13 (0.79)	−0.93 (1.02)	< 0.001
Gestational Age (weeks)	26.4 (1.5)	27.3 (1.2)	< 0.001
Antenatal Corticosteroids	59 (89%)	76 (92%)	0.650
Maternal Smoking in Pregnancy	17 (28%)	11 (14%)	0.036
Singleton Delivery	57 (86%)	66 (79%)	0.220
Surfactant	66 (100%)	79 (94%)	0.067
Supplementary Oxygen at 36wks	41 (62%)	37 (44%)	0.033
Postnatal corticosteroids	18 (27%)	23 (27%)	0.990
HFOV	37 (56%)	40 (48%)	0.310
Age at testing (years)	17.9 (0.8)	18.0 (0.8)	0.510
Weight at testing (kg)	63.0 (16.1)	64.0 (15.9)	0.710
Height at testing (cm)	166.4 (8.7)	167.7 (9.1)	0.600
Active Smoker	9 (14%)	9 (11%)	0.580
History of Asthma	18 (28%)	23 (28%)	> 0.990

*Note:* Data are demonstrated as n (%) or the mean (SD).

**Table 2:** Demographics by maternal labor status.

	Maternal Labor	Mother not in Labor	P value
**Number**	**93**	**57**	
Female	46 (49%)	34 (60%)	0.230
Birthweight (gram)	939 (214)	842 (216)	0.020
Birthweight z‐score	−0.23 (0.79)	−1.14 (1.08)	< 0.001
Gestational Age (weeks)	26.7 (1.5)	27.3 (1.2)	0.005
Antenatal Corticosteroids	82 (88%)	53 (95%)	0.200
Maternal Smoking in Pregnancy	22 (26%)	6 (11%)	0.036
Singleton Delivery	73 (78%)	50 (88%)	0.160
Surfactant	93 (100%)	52 (91%)	0.007
Supplementary Oxygen at 36w	43 (46%)	29 (51%)	0.580
Postnatal corticosteroids	22 (24%)	19 (33%)	0.200
HFOV	51 (55%)	26 (46%)	0.270
Age at testing (years)	18.0 (0.8)	18.0 (0.8)	0.930
Weight at testing (kg)	64.7 (16.1)	61.7 (15.7)	0.280
Height at testing (cm)	167.4 (8.7)	166.6 (9.2)	0.730
Active Smoker	13 (14%)	5 (9%)	0.320
History of Asthma	26 (28%)	15 (27%)	0.850

*Note*: Data are demonstrated as n (%) or the mean (SD).

**Table 3:** Lung function by type of delivery.

	Vaginal Delivery	Cesarean	Difference (95% CI)	Adjusted Difference^a^	P value
			(caesarian ‐ vaginal)	(95% CI)	
FEF_75_	−0.81 (1.35)	−1.15 (1.18)	−0.32 (−0.74, 0.11)	−0.51 (−0.99, −0.04)	0.040
FEF_50_	−0.84 (1.16)	−1.08 (0.97)	−0.20 (−0.55, 0.15)	−0.37 (−0.77, 0.03)	0.070
FEF_25_	−0.47 (1.17)	−0.74 (1.14)	−0.25 (−0.64, 0.13)	−0.42 (−0.86, 0.03)	0.070
FEF_25‐75_	−1.28 (1.36)	−1.58 (1.21)	−0.27 (−0.70, 0.16)	−0.42 (−0.89, 0.06)	0.096
FEV_1_	−1 (1.31)	−0.99 (1.33)	0.04 (−0.48, 0.40)	−0.15 (−0.66, 0.35)	0.560
FVC	−0.42 (1.30)	−0.09 (1.36)	0.25 (−0.20, 0.70)	0.2 (−0.34, 0.74)	0.480
FEV_1_/FVC	−0.91 (1.19)	−1.30 (1.21)	−0.33 (−0.73, 0.07)	−0.48 (−0.95, 0)	0.055
PEF	−0.41 (1.16)	−0.47 (1.05)	−0.06 (−0.42, 0.30)	−0.25 (−0.67, 0.18)	0.270
DLCO	−1.02 (1.20)	−0.99 (1.02)	0.02 (−0.36, 0.40)	−0.048 (−0.41, 0.50)	0.840
DLCO/VA	−1.93 (1.03)	−2.18 (0.77)	−0.26 (−0.56, 0.05)	−0.27 (−0.63, 0.09)	0.160
FRC_PLETH_	0.45 (1.36)	0.71 (1.32)	−0.27 (−0.17, 0.70)	−0.1 (−0.41, 0.63)	0.690
FRC_HE_	0.37 (1.91)	0.81 (2.12)	0.45 (−0.26, 1.15)	0.05 (−0.78, 0.88)	0.910
RV	1.02 (1.40)	1.06 (1.30)	−0.04 (−0.39, 0.48)	0.0009 (−0.49, 0.50)	0.990
TLC	0.75 (1.19)	0.90 (1.16)	0.14 (−0.26, −0.54)	−0.04 (−0.52, 0.44)	0.870
Resistance (5_Hz_)	−0.10 (1.20)	−0.18 (1.06)	−0.09 (−0.47, 0.28)	0.26 (−0.16, 0.68)	0.230
Resistance (20_Hz_)	0.39 (1.06)	0.25 (0.97)	−0.14 (−0.47, 0.19)	0.1 (−0.27, 0.48)	0.590
LCI	9.17 (1.62)	9.30 (1.58)	0.18 (−0.41, 0.77)	0.25 (−0.43, 0.93)	0.480
Exercise Capacity	1054 (258)	1085 (241)	15.4 (−74.2, 105.0)	−6.5 (−107.1, 94.1)	0.900

*Note:* Data are presented as mean (SD) *n* = 150 (max).

^a^ Adjusted model includes the following covariates: birthweight z‐score, gestational age, maternal smoking in pregnancy (yes/no), postnatal surfactant (yes/no), participant age at assessment and allows for nonindependence of multiple births.

Four measures of lung function (FEF_75_, FEF_50_, FEF_25‐75_, and LCI) differed significantly between those whose mother was and was not in labor at their birth with a difference of around one‐half z‐score (approximately one standard deviation) for the FEF measures and approximately one‐half standard deviation for LCI (Table 4).

**Table 4:** Lung function by labor status.

	Maternal Labor	Mother not in Labor	Difference (95% CI)	Adjusted Difference^a^	P value
			(labor – no labor)	(95% CI)	
FEF_75_	−0.82 (1.30)	−1.29 (1.17)	0.44 (0.01, 0.86)	0.50 (0.02, 0.99)	0.047
FEF_50_	−0.82 (1.10)	−1.22 (0.96)	0.39 (0.04, 0.74)	0.45 (−0.05, 0.85)	0.032
FEF_25_	−0.46 (1.17)	−0.88 (1.10)	0.35 (−0.03, 0.74)	0.38 (−0.07, 0.84)	0.100
FEF_25‐75_	−1.24 (1.31)	−1.78 (1.17)	0.49 (0.06, 0.92)	0.53 (0.05, 1.01)	0.037
FEV_1_	−0.86 (1.29)	−1.22 (1.35)	0.32 (−0.13, 0.77)	0.42 (−0.08, 0.93)	0.110
FVC	−0.21 (1.28)	−0.27 (1.44)	0.018 (−0.44, 0.47)	0.18 (−0.36, 0.72)	0.530
FEV_1_/FVC	−0.96 (1.19)	−1.39 (1.21)	0.42 (0.02, 0.82)	0.39 (−0.09, 0.88)	0.120
PEF	−0.32 (1.09)	−0.64 (1.08)	0.22 (−0.14, 0.58)	0.33 (−0.10, 0.76)	0.140
DLCO	−1.02 (1.10)	−0.97 (1.10)	−0.05 (−0.44, 0.33)	−0.16 (−0.61, 0.29)	0.500
DLCO/VA	−2.00 (0.98)	−2.18 (0.76)	0.15 (−0.16, 0.46)	0.04 (−0.32, 0.40)	0.830
FRC_PLETH_	0.46 (1.30)	0.82 (1.39)	−0.36 (−0.8, 0.08)	−0.14 (−0.66, 0.38)	0.610
FRC_HE_	0.50 (1.98)	0.78 (2.13)	−0.23 (−0.95, 0.49)	−0.11 (−0.74, 0.95)	0.810
RV	0.91 (1.28)	1.26 (1.40)	−0.35 (−0.79, 0.09)	−0.27 (−0.77, 0.22)	0.290
TLC	0.80 (1.15)	0.89 (1.20)	−0.08 (−0.49, 0.32)	0.16 (−0.32, 0.65)	0.520
Resistance (5 _Hz_)	−0.04 (1.21)	−0.32 (0.95)	0.28 (−0.09, 0.65)	0.13 (−0.28, 0.55)	0.540
Resistance (20 _Hz_)	0.43 (1.03)	0.12 (0.94)	0.31 (−0.02, 0.64)	0.15 (−0.23, 0.53)	0.440
LCI	8.99 (1.54)	9.63 (1.59)	−0.63 (−1.21, −0.05)	−0.72 (−1.35, −0.08)	0.033
Exercise Capacity	1094 (266)	1044 (217)	38.1 (−49.9, 126.1)	65.1 (−33.0, 163.2)	0.190

*Note:* Data are presented as mean (SD) *n* = 150 (max).

^a^ Adjusted model includes the following covariates: birthweight z‐score, gestational age, maternal smoking in pregnancy (yes/no), postnatal surfactant (yes/no), participant age at assessment and allows for nonindependence of multiple births.

Twenty percent of those whose mothers were not in labor reported symptoms of wheeze compared to 13% of those whose mothers were in labor, but this was not statistically significant (*p* = 0.24). Overall, none of the measures of exercise or symptoms/medication differed significantly by delivery or labor status (Tables 5, 6).

**Table 5:** Exercise and respiratory outcomes by delivery type.

	N	Vaginal Delivery	Cesarean	P value^a^	P value^b^
				(unadjusted model)	(adjusted model)
Exercise distance test	126			0.480	0.290
< 1000 m		22 (45%)	28 (36%)		
1000–1249 m		15 (31%)	29 (38%)		
1250–1500 m		12 (24%)	20 (26%)		
Self‐reported exercise	143			0.410	0.790
None		16 (26%)	26 (32%)		
Up to 1 h/day		31 (51%)	41 (50%)		
More than 1 h/day		14 (23%)	15 (18%)		
Symptoms/medication					
Any wheeze in last 12 months	148	13 (20%)	11 (13%)	0.270	0.240
Current asthma	149	9 (14%)	5 (6%)	0.110	0.150
Inhaler use	149	9 (14%)	4 (5%)	0.077	0.060

^a^ Model is adjusted for multiple births via robust standard error.

^b^ Model is adjusted for birthweight z‐score and multiple births via robust standard error.

**Table 6:** Self‐reported symptoms and exercise tolerance by labor status.

	N	Maternal Labor	Mother not in Labor	P value^a^	P value^b^
				(unadjusted model)	(adjusted model)
Exercise distance test	126			0.520	0.690
< 1000 m		29 (40%)	21 (40%)		
1000–1249 m		22 (30%)	22 (42%)		
1250–1500 m		22 (30%)	10 (19%)		
Self‐reported exercise	143			0.029	0.110
None		22 (25%)	20 (36%)		
Up to 1 h/day		43 (49%)	29 (53%)		
More than 1 h/day		23 (26%)	6 (11%)		
Symptoms/medication					
Any wheeze in last 12 months	148	18 (20%)	6 (11%)	0.160	0.130
Current asthma	149	12 (13%)	2 (4%)	0.070	0.080
Inhaler use	149	11 (12%)	2 (4%)	0.130	0.098

^a^ Model is adjusted for multiple births via robust standard error.

^b^ Model is adjusted for birthweight z‐score and multiple births via robust standard error.

## 4 Discussion

Our study demonstrated a significant difference in small airway lung function between prematurely born young adults who were born to mothers in labor compared to those whose mothers were not in labor, while only one significant difference was observed according to mode of delivery. Differences observed were of the order of one half a standard deviation which is sizable, suggesting that the participant may be vulnerable to later respiratory morbidity. Our findings may explain the heterogeneity of evidence of the impact of mode of delivery on respiratory morbidity in later life, as labor status was not considered in many previous studies [15, 16, 17, 18, 19, 22]. Small airway function tests such as FEF_75_ have been posited as early indicators of small airway disease where FEV_1_ and FVC values remain normal [48, 49]. Lower FEF_75_ and FEF_25‐75_ values are linked to a number of diseases with small airway involvement [50]. Similarly, increased LCI values can indicate ventilatory homogeneity and thus demonstrate abnormalities in small airway function [51]. A possible explanation might be that in term born infants, those whose mothers were in labor established an earlier FRC and thus may have had better lung function in the perinatal period [11]. If this were the case for extremely prematurely born infants, those born after maternal labor may have had less exposure to the injurious effects of high levels of respiratory support [52]. We demonstrated that infants born to mothers not in labor were less likely to receive surfactant than those born to mothers in labor (*p* = 0.007). It is possible that the higher mean gestation in the nonlabour group resulted in some clinical scenarios where surfactant was not indicated, and this was taken into consideration in the analysis.

Our data on small airway testing is broadly similar to other published evidence of lung function testing in ex‐preterm adult cohorts. Yang et al. demonstrated that in an ex‐preterm cohort with a mean age of 28.5 (SD 1.1), the FEF_25‐75_ z‐score was −1.29 (SD 1.28) [25]. Similarly, Vollsaeter et al. reported FEF_25‐75_ z‐scores of −1.32 (95% CI −1.66, −0.99) at 25 years of age in an ex‐preterm cohort [53]. These values were significantly worse than term‐born controls. It is possible that individuals who have poorer small airway function may have early changes of respiratory disease despite normal large airway function, and that larger airway dysfunction may develop in the future.

Prematurity has been shown to have a negative effect on the immune system with one study showing decreased activation of neutrophils associated with premature birth [54]. In addition, prematurity is associated with a decrease in levels of galectin‐3, an important modulator and primer of the immune system [55]. An alternative explanation then for our results is that exposure to bacteria in the birth canal stimulates the neonatal immune system, conveying protection in infancy. Several studies have demonstrated that a lack of exposure to microbes in early life is associated with the development of asthma in later life [56, 57].

Our study has several strengths and some limitations. We measured lung function in a large number of extremely prematurely born individuals at 16–19 years of age and thus were able to tease out the effects of the mode of delivery from whether the mothers were in labor. Our analysis adjusted for maternal and neonatal factors—but it is possible that differences in birthweight may have accounted for some of our findings. We had formally assessed the smoking habits of the individuals, a known risk factor for impaired lung function in young adults born prematurely [24, 58]. We did not have data regarding ventilatory settings in the first week after birth to confirm our hypothesis that those born after maternal labor might have had better lung function after birth, but other evidence would suggest that is correct [59, 60]. Non‐respondents were more likely to be nonwhite, have cranial ultrasound abnormalities, mothers who smoked, or have had pulmonary hemorrhages—this may have impacted on our findings. We also acknowledge that FEF_75_ or FEF_25‐75_ measurements are not recognized by ERS/ATS technical guidelines for the identification of small airway disease, due to concerns about poor reproducibility. We have reported z‐scores to try and mitigate this.

In conclusion, we have demonstrated that very prematurely born individuals born after maternal labor demonstrated evidence of better small airway function at 16–19 years than those who were not, but mode of delivery was not associated with differences in lung function in young adults. It is possible that we have identified a cohort of individuals within a high‐risk group who have early signs of small airway disease, despite relatively normal lung function testing overall, and are at higher long‐term risk of respiratory morbidity. Clinicians should be aware that the absence of maternal labor may adversely influence the long‐term respiratory health of extremely preterm infants. It would be important to further follow this cohort to determine if the small airway changes are subsequently associated with other lung function abnormalities.

## Author Contributions

**Sean Armstrong:** writing–original draft, writing–review and editing, formal analysis, visualization, data curation. **Christopher Harris:** investigation, writing–original draft, writing–review and editing, formal analysis, data curation, conceptualization. **Mohadeseh Kazemi:** methodology, formal analysis, data curation. **Alan Lunt:** investigation, visualization, resources. **Janet Peacock:** writing–review and editing, formal analysis; data curation, methodology. **Anne Greenough:** conceptualization, investigation, funding acquisition, writing–review and editing, supervision, project administration.

## Ethics Statement

Ethical approval for UKOS was granted by the Thames Multicentre Research Ethics Committee and for the follow‐up study by the North‐East–Tyne & Wear South Research Ethics Committee.

## Conflicts of Interest

The authors declare no conflicts of interest.

## Supporting information

Supporting information.

## Data Availability

The data that support the findings of this study are available on request from the corresponding author. The data are not publicly available due to privacy or ethical restrictions.
