# Supplementary material for: Labor Status at Delivery and Lung Function in Extremely Prematurely Born Young Adults
Source: Pediatr Pulmonol. 2024 Dec 16;60(1):e27440. doi: 10.1002/ppul.27440 (PMC11748109; doi:10.1002/ppul.27440)
Supplement: Supplementary file 1 — Supporting information. [file PPUL-60-0-s001.docx]

**Supplement table** E-Table 1 Comparison of baseline characteristics between children recruited or not recruited

The data are presented as the mean (SD) or number (%) unless specified

|  | **Assessed at age 16-19 yrs** | **Non-respondents at age 16-19 yrs** | **P value** |
| --- | --- | --- | --- |
| **N** | 159 | 638 |  |
| Male (%) | 77 (48) | 351 (55) | 0.160 |
| Mother’s ethnicity (%) |  |  | <0.001 |
| White | 139 (87) | 497 (78) |  |
| Black | 15 (9) | 55 (9) |  |
| Other | 5 (3) | 83 (13) |  |
| Birthweight (g) | 894 (218) | 843 (218) | 0.009 |
| Birthweight z-score | -0.6 (1.0) | -0.6 (1.0) | 0.860 |
| Gestational age, weeks | 26.9 (1.5) | 26.4 (1.5) | <0.001 |
| Multiple birth (%) | 29 (18) | 161 (25) | 0.080 |
| Surfactant given (%) | 154 (97) | 615 (96) | >0.990 |
| Mother smoked in pregnancy (%) | 30 (20) | 170 (29) | 0.034 |
| Systemic steroids given prior to extubation (%) | 49 (31) | 150 (29) | 0.695 |
| Oxygen dependency at 36 weeks postmenstrual age (%) | 86 (54) | 257 (57) | 0.552 |
| Oxygen dependency at 28 days (%) | 122 (77) | 384 (82) | 0.176 |
| Oxygen dependent at discharge (%) | 37 (24) | 85 (20) | 0.370 |
| Ultrasound abnormality (%) | 14 (9) | 115 (18) | 0.005 |
| HFOV (%) | 81 (51) | 319 (50) | 0.900 |
| Patent ductus arteriosus (%) | 44 (28) | 222 (35) | 0.110 |
| Pulmonary hemorrhage (%) | 7 (4) | 92 (15) | 0.001 |
| Airleak (%) | 21 (13) | 115 (18) | 0.190 |
